# ^68^Ga-Labeled Cyclic NGR Peptide for MicroPET Imaging of CD13 Receptor Expression

**DOI:** 10.3390/molecules190811600

**Published:** 2014-08-05

**Authors:** Yahui Shao, Wansheng Liang, Fei Kang, Weidong Yang, Xiaowei Ma, Guiyu Li, Shu Zong, Kai Chen, Jing Wang

**Affiliations:** 1 Department of Nuclear Medicine, Xijing Hospital, The Fourth Military Medical University, Xi’an 710032, China; 2 Department of Nuclear Medicine, General Hospital of Jinan Military Area Command, Jinan 250031, China; 3 Department of Nuclear Medicine, Lanzhou General Hospital of Lanzhou Military Area Command, Lanzhou 730050, China; 4 Molecular Imaging Center, Department of Radiology, Keck School of Medicine, University of Southern California, Los Angeles, CA 90033, USA

**Keywords:** microPET imaging, NGR peptide, CD13, tumor angiogenesis, ^68^Ga labeling

## Abstract

Peptides containing the asparagines-glycine-arginine (NGR) motif have been identified as specific ligands binding to CD13/aminopeptidase N (APN) receptor, a tumor neovascular biomarker. In this study, we synthesized a novel NGR-containing peptide (NOTA-G_3_-NGR), and labeled NOTA-G_3_-NGR with ^68^Ga (t_1/2_ = 67.7 min). The resulting ^68^Ga-NOTA-G_3_-NGR peptide was subject to *in vitro* and *in vivo* characterization. The microPET imaging results revealed that the ^68^Ga-NOTA-G_3_-NGR peptide exhibits rapid and specific tumor uptake, and high tumor-to-background contrast in a subcutaneous HT-1080 fibrosarcoma mouse model. We concluded that the ^68^Ga-NOTA-G_3_-NGR peptide has potential in the diagnosis of CD13-targeted tumor angiogenesis.

## 1. Introduction

Positron emission tomography (PET) is one of the most sensitive molecular imaging modalities, which is capable of noninvasive visualization, characterization, and measurement of biological processes in living subjects [1,2,3]. PET imaging probes labeled with an appropriate positron emitter have been used to examine various diseases at the cellular, subcellular, or even molecular level [4,5,6]. Numerous molecules, such as peptides [7,8,9,10], antibodies [11], nucleic acids [12], and small-molecule ligands [13,14] have been developed as PET probes. Among these probes, radiolabeled peptides have attracted significant attention due to their favorable properties, including rapid tissue penetration, fast clearance, low immunogenicity, high affinity to targets, good *in vivo* stability and integrity, and relatively easy production [5,9,15,16]. Enormous progress has been made in peptidic PET probes for diagnostic applications during the last decade [5,9,15,17].

The peptides containing asparagine-glycine-arginine (NGR) motif are ones of the most distinguished peptides discovered by phage display technology [18,19]. Further studies revealed that the NGR-containing peptides specifically bind to tumor neovascular CD13/APN (aminopeptidase N) receptor, which is selectively overexpressed on tumor vasculature and some tumor cells [18,19]. Among all tested peptides in phage display peptide library, the NGR peptide showed the greatest tumor selectivity [20], which is about three-fold higher than that of the arginine-glycine-aspartic acid (RGD) peptide [21].

Recently, we have successfully synthesized a series of NGR-containing peptides, and labeled them with either ^64^Cu for PET [8] or ^99m^Tc for SPECT [22]. Our preclinical results demonstrated that these radiolabeled NGR peptides can be used as molecular probes for specific imaging of CD13 receptor expression in tumors. Overall, PET imaging provides higher spatial resolution compared to SPECT. Although ^64^Cu has attracted considerable interest as a PET radionuclide due to its favorable decay half-life [10,23], production of ^64^Cu requires a biomedical cyclotron, which is relatively expensive. As compared to ^64^Cu, ^68^Ga has higher positron abundance, and it can be obtained from a ^68^Ge/^68^Ga generator system which is simple in use [24]. For ^68^Ga labeling, one of commonly used chelators is 1,4,7-triazacyclononane-N,N',N''-triacetic acid (NOTA). A sizable body of evidence suggests that the labeling procedure of ^68^Ga-NOTA system is efficient, reproducible, and affordable, which is suitable for kit formulation [24]. To this end, a NOTA-conjugated NGR-containing peptide (NOTA-G_3_-NGR) was successfully prepared and labeled with ^68^Ga to afford ^68^Ga-NOTA-G_3_-NGR. The *in vitro* stability, lipophilicity, binding affinity, and tumor cell uptake of ^68^Ga-NOTA-G_3_-NGR were subsequently investigated. The ability of using ^68^Ga-NOTA-G_3_-NGR to image CD13 receptor expression *in vivo* by PET was assessed in subcutaneous CD13-positive HT-1080 fibrosarcoma and HT-29 colon adenocarcinoma mouse xenografts. CD13 specificity of ^68^Ga-NOTA-G_3_-NGR was evaluated by *in vivo* blocking studies.

## 2. Results and Discussion

### 2.1. Chemistry and Radiochemistry

**Scheme 1:**
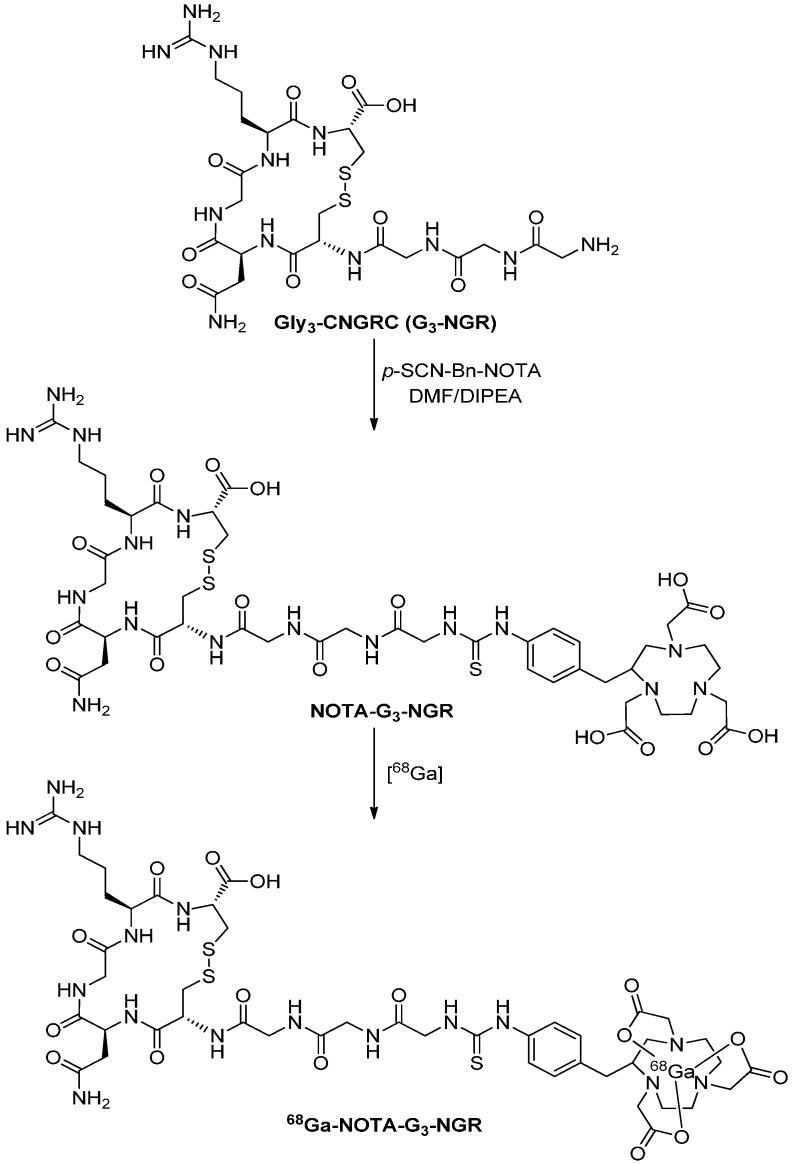
Synthetic scheme of ^68^Ga-NOTA-G_3_-NGR peptide.

Preparation of NOTA-G_3_-NGR and radiosynthesis of ^68^Ga-NOTA-G_3_-NGR are shown in Scheme 1. The conjugation of Gly_3_-CNGRC (G_3_-NGR) peptide with *p*-SCN-Bn-NOTA provided the NOTA-G_3_-NGR peptide in a yield of 77%. The NOTA-G_3_-NGR peptide was purified using HPLC to achieve a purity of >95% (Figure S1). The NOTA-G_3_-NGR peptide was characterized by mass spectrometry (ESI-MS: *m/z* 1172.00 for [M+H]^+^ (C_44_H_67_N_16_O_16_S_3_); calc. 1171.40) (Figure S2). ^68^Ga labeling was achieved within 30 min. The decay-corrected yields ranged from 95% to 98%, and the radiochemical purity was >99% after purification (Figure S3). The probe was used immediately after formulation. The specific radioactivity of ^68^Ga-NOTA-G_3_-NGR was estimated to be 13–16 MBq/nmol before the animal studies.

### 2.2. Octanol-Water Partition Coefficient

The octanol-water partition coefficient of ^68^Ga-NOTA-G_3_-NGR was determined to be −2.25 ± 0.17, suggesting that ^68^Ga-NOTA-G_3_-NGR peptide is quite hydrophilic.

### 2.3. In Vitro Stability

**Figure 1:**
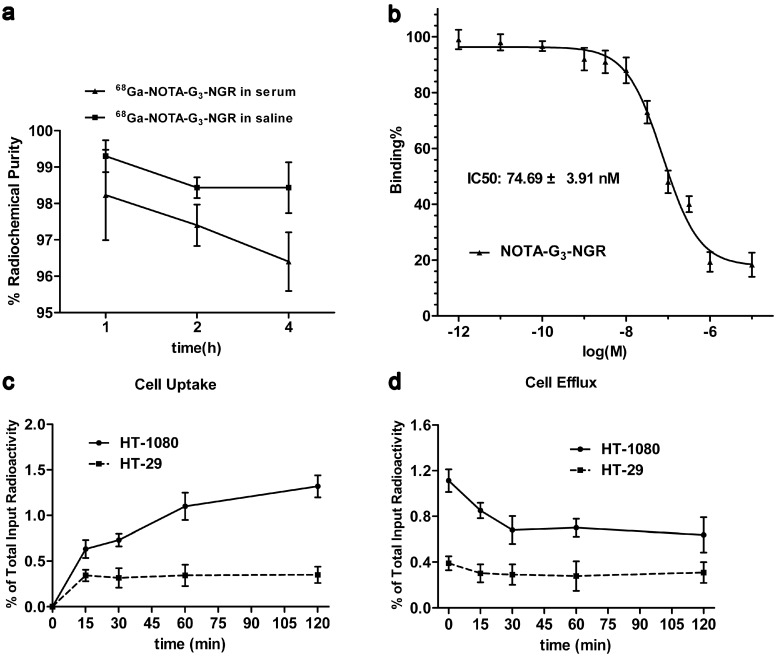
(**a**) *In vitro* stability of ^68^Ga-NOTA-G_3_-NGR in saline at room temperature and in human serum at 37 °C for 1, 2, and 4 h; (**b**) *In vitro* inhibition of ^125^I-NGR binding to CD13 receptor by NOTA-G_3_-NGR in HT-1080 cells. The IC_50_ value of NOTA-G_3_-NGR was calculated to be 74.69 ± 3.91 nM (*n* = 3); (**c**) Cell uptake of ^68^Ga-NOTA-G_3_-NGR in HT-1080 and HT-29 cells (*n* = 3, mean ± SD); (**d**) Cell efflux of ^68^Ga-NOTA-G_3_-NGR in HT-1080 and HT-29 cells (*n* = 3, mean ± SD).

The *in vitro* stability of ^68^Ga-NOTA-G_3_-NGR in saline at room temperature and in fresh human serum at 37 °C is shown in Figure 1a. After 4 h incubation, >98% of ^68^Ga-NOTA-G_3_-NGR in saline at room temperature and >96% of ^68^Ga-NOTA-G_3_-NGR at 37 °C in human serum remained intact, indicating that ^68^Ga-NOTA-G_3_-NGR is stable *in vitro*.

### 2.4. Cell-Based Binding Assay

*In vitro* CD13 binding affinity and specificity of NOTA-G_3_-NGR were tested by a cell-based competitive assay. The binding of ^125^I-NGR to CD13 receptors in HT-1080 cells can be inhibited by NOTA-G_3_-NGR in a dose-dependent manner (Figure 1b). The concentrations of NOTA-G_3_-NGR peptide were at a range from 10^−12^ to 10^−5^ M. The IC_50_ value of NOTA-G_3_-NGR was calculated to be 74.69 ± 3.91 nM.

### 2.5. Cell Uptake and Efflux

Western blot analysis showed that CD13 receptors are overexpressed in HT-1080 cells, but not in HT-29 cells (Figure S4). To determine specific cell binding and retention properties, ^68^Ga-NOTA-G_3_-NGR was incubated with HT-1080 and HT-29 cells, respectively. The results demonstrated that ^68^Ga-NOTA-G_3_-NGR could bind to CD13-positive HT-1080 cells, but not CD13-negative HT-29 cells. At the first hour incubation, 1.10% ± 0.10% of ^68^Ga-NOTA-G_3_-NGR uptake in HT-1080 cells was determined. After 2 h incubation, the uptake of ^68^Ga-NOTA-G_3_-NGR in HT-1080 cells reached the maximum (1.32% ± 0.12%) (Figure 1c). For cell efflux study, approximately 0.7% of ^68^Ga-NOTA-G_3_-NGR retention in HT-1080 cells was determined after 2 h incubation (Figure 1d). In contrast, cellular uptake and retention of ^68^Ga-NOTA-G_3_-NGR in HT-29 cells were determined at the minimal levels of input radioactivity after incubation. The maximum uptake of ^68^Ga-NOTA-G_3_-NGR in HT-29 cells after 2 h incubation was about 0.35% ± 0.10% (Figure 1c), and the retention value of ^68^Ga-NOTA-G_3_-NGR in HT-29 cells after 2 h incubation was determined to be 0.31% ± 0.10% (Figure 1d).

### 2.6. MicroPET Imaging

**Figure 2:**
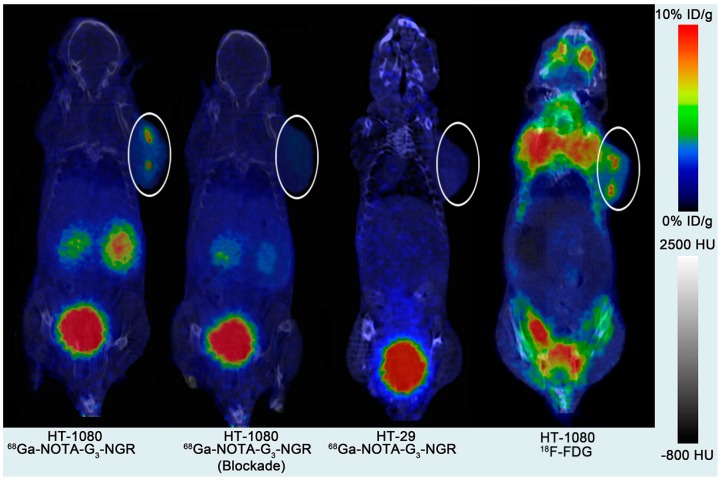
Decay-corrected coronal image slices at 1 h pi of ^68^Ga-NOTA-G_3_-NGR in HT-1080 and HT-29 tumor xenografts with and without co-injection of non-radiolabeled NOTA-G_3_-NGR peptide as a blocking agent. As a comparison, decay-corrected coronal image slice at 1 h pi of ^18^F-FDG in HT-1080 tumor xenografts was also shown. Tumors are indicated using circles.

Static microPET scans were performed at 0.5, 1, and 2 h post-injection (pi) of ^68^Ga-NOTA-G_3_-NGR in HT-1080 and HT-29 tumor xenografts (*n* = 5/group). The CD13-positive HT-1080 tumors were clearly visible with good tumor-to-background contrast at all measured time points. For CD13-negative HT-29 tumors, ^68^Ga-NOTA-G_3_-NGR exhibited minimal tumor uptake. Representative decay-corrected coronal image slices are shown in Figure 2. As a comparison, microPET images of ^18^F-FDG were also acquired in HT-1080 tumor xenografts. The mice were fasted for 6 h and then injected with 3.7 MBq of ^18^F-FDG. Static PET scans (10 min) and helical CT scans were acquired at 1 h pi (Figure 2).

**Figure 3:**
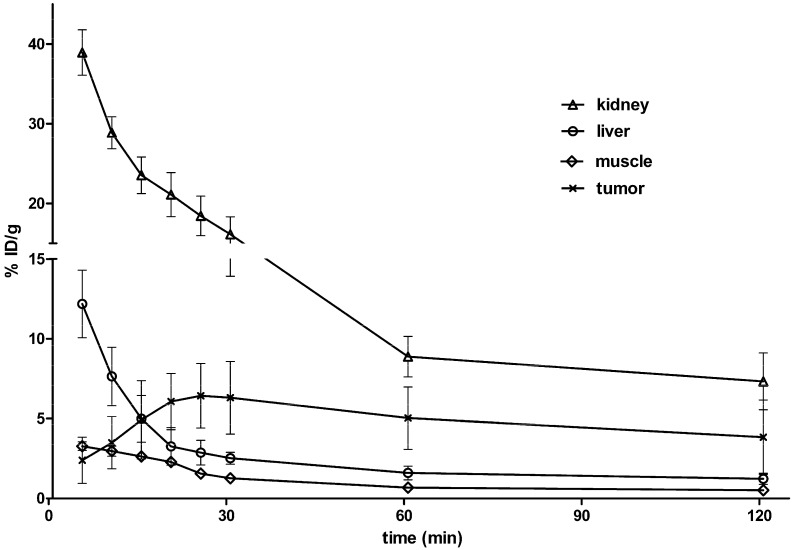
Time-activity curves from quantitative microPET imaging analysis of ^68^Ga-NOTA-G_3_-NGR.

The tumor and major organ uptake levels of ^68^Ga-NOTA-G_3_-NGR were calculated by measuring region of interest (ROI) encompassing the entire tissue or organ in the coronal orientation of the microPET images. The time-activity curves (30-min decay-corrected dynamic scans followed by static scans) of ^68^Ga-NOTA-G_3_-NGR in HT-1080 tumor xenografts are shown in Figure 3. Uptake values are shown as mean %ID/g (percentage of injected dose per gram of tissue) ± SD (*n* = 5/group). HT-1080 tumor uptake of ^68^Ga-NOTA-G_3_-NGR was determined to be 6.30% ± 2.27%, 5.03% ± 1.95%, and 3.84% ± 2.32% ID/g at 0.5, 1, and 2 h pi, respectively. ^68^Ga-NOTA-G_3_-NGR excreted rapidly through the kidneys, and the probe accumulation in most other normal organs at 2 h pi was very low.

**Figure 4:**
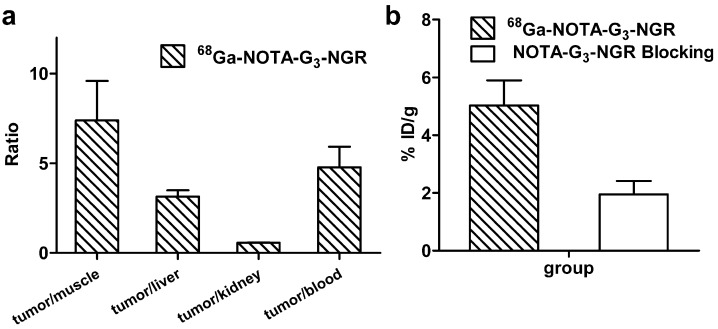
(**a**) The T/NT ratios at 1 h pi quantified by microPET imaging; (**b**) The tumor uptake of ^68^Ga-NOTA-G_3_-NGR peptide with the non-radiolabeled peptide *vs.* without the non-labeled peptide at 1 h pi quantified by microPET imaging.

The tumor-to-normal tissue (T/NT) ratios of ^68^Ga-NOTA-G_3_-NGR at 1 h pi were calculated (Figure 4a). The ratios of HT-1080 tumor uptake to muscle, liver, kidney, and blood at 1 h pi were 7.39 ± 2.20, 3.14 ± 0.35, 0.57 ± 0.02, and 4.78 ± 1.14, respectively. Co-injection of NOTA-G_3_-NGR peptide (20 mg/kg) led to significant reduction of HT-1080 tumor uptake. The HT-1080 tumor uptake of ^68^Ga-NOTA-G_3_-NGR at 1 h pi was 5.03% ± 1.95% ID/g in non-blocking group, whereas 1.95% ± 1.05% ID/g was determined in the blocking group (co-injection with unlabeled NOTA-G_3_-NGR peptide) (Figure 4b). The immunohistochemistry staining of HT-1080 tumor slices further confirmed that CD13 receptors were indeed overexpressed in HT-1080 tumors (Figure S5).

### 2.7. Biodistribution Studies

**Figure 5:**
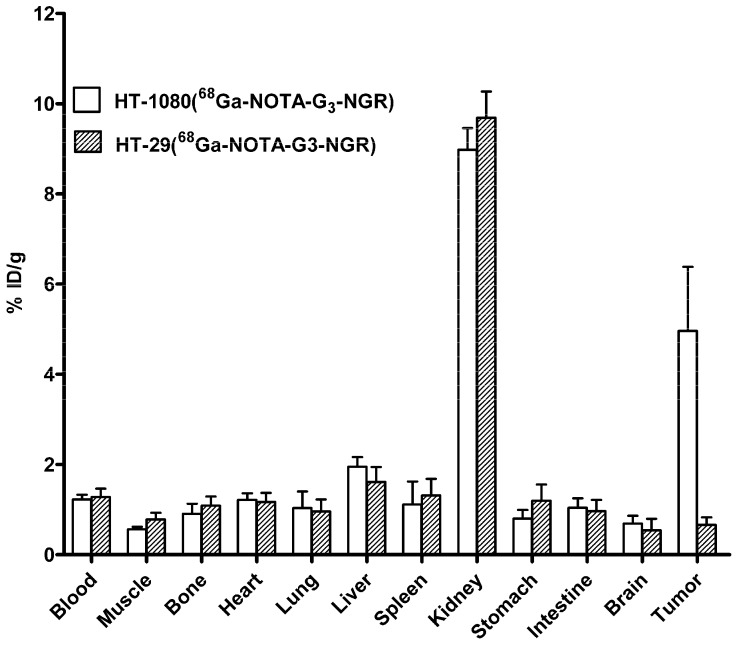
Decay-corrected biodistribution at 1 h post-injectioin of ^68^Ga-NOTA-G_3_-NGR peptide in HT-1080 and HT-29 tumor xenografts.

*Ex vivo* biodistribution study was performed at 1 h pi of ^68^Ga-NOTA-G_3_-NGR using nude mice bearing HT-1080 or HT-29 tumors (*n* = 5, mean ± SD). The results were consistent with the quantitative analyses of microPET imaging. As shown in Figure 5, the HT-1080 tumor uptake of ^68^Ga-NOTA-G_3_-NGR reached 4.96% ± 3.18% ID/g, whereas HT-29 tumor uptake remained at a minimal level (0.88% ± 0.68% ID/g). In addition, ^68^Ga-NOTA-G_3_-NGR exhibited minimal uptake in most normal organs, except for kidneys.

## 3. Experimental Section

### 3.1. General

All commercially obtained chemicals were of analytical grade and used without further purification. Cyclic NGR peptide [GGGCNGRC; disulfide Cys:Cys = 4–8] was purchased from CS Bio Company, Inc. (Menlo Park, CA, USA). The *p*-SCN-Bn-NOTA chelator was obtained from Macrocyclics Inc. (Dallas, TX, USA). Mass spectrometric data were recorded using a Thermo-Electron Finnigan LTQ mass spectrometer equipped with an electrospray ionization source (Thermo Scientific, Waltham, MA, USA). ^68^Ga was obtained from a ^68^Ge/^68^Ga generator (ITM Isotopen Technologien München AG, Garching, Germany) and eluted with 4 mL of 0.05 M HCl. Radiochemical purity (RCP) was measured by radio-TLC which was performed on silica gel-coated plastic sheets (Polygram SIL G, Macherey-Nagel, Bethlehem, PA, USA) with sodium citrate (0.1 M, pH = 5) as developing solvent. The radio-TLC results were recorded using Bioscan Mini-scan (Washington, DC, USA) and Allchrom Plus software.

### 3.2. Synthesis of NOTA-G_3_-NGR

The *p*-SCN-Bn-NOTA chelator (3 mg, 4.16 μmol) in 25 μL of dimethyl sulfoxide (DMSO) was added to a vial containing 2.56 mg (4.58 μmol) of Gly_3_-CNGRC (G_3_-NGR) peptide and 20 μL of diisopropylethylamine in 0.2 mL of *N*,*N*-dimethylformamide (DMF). After 1 h, the reaction was quenched with 20 μL of acetic acid in 0.5 mL of water. The reaction mixture was purified with a semi-preparative HPLC running a linear gradient starting from 95% A (0.1% TFA in water) and 5% B (0.1% TFA in acetonitrile) for 5 min and increasing to 65% B at 35 min with a flow rate of 12 mL/min. The fractions containing the desired product were collected and lyophilized to give 3.75 mg (77%) of white powder.

### 3.3. ^68^Ga Labeling and Formulation

The NOTA-G_3_-NGR peptide was labeled according to a previously described method [25] with slight modifications. In brief, the NOTA-G_3_-NGR peptide (15 nmol) was dissolved in 500 μL of 0.1 M sodium acetate buffer and incubated with 1295 MBq of ^68^Ga for 10 min at 42 °C. After cooling to room temperature, 30 μL of sodium acetate buffer (1.25 M) was added to the mixture. The mixture was then passed through a Sep-Pak C18 cartridge (Waters Corporation, Vienna, Austria) and washed with 5 mL of water. ^68^Ga-NOTA-G_3_-NGR was eluted with 2 mL of 50% ethanol/saline, and concentrated by rotary evaporation. The product was re-dissolved in 2 mL of water, and passed through a 0.22 μm Millipore filter into a sterile dose vial for use in the following experiments.

### 3.4. Octanol–Water Partition Coefficient

The octanol–water partition coefficient value was determined by measuring the distribution (logD) of radioactivity in octanol and PBS. Approximately 5 KBq of ^68^Ga- NOTA-G_3_-NGR (2 μL) was added to a vial containing 0.5 mL of octanol and 0.5 mL of PBS (pH = 7.4). The mixture was then vortexed vigorously for 15 min. Subsequently, the mixture was centrifuged at 12,500 rpm for 5 min to ensure the complete separation of layers. Aliquots of the aqueous and octanol layers were collected, measured in a gamma counter (Beijing PET CO., Ltd., Beijing, China). The octanol–water partition coefficient value was then calculated (*n* = 5).

### 3.5. In Vitro Stability

The stability of ^68^Ga-NOTA-G_3_-NGR was tested by mixing 3.7MBq of ^68^Ga-NOTA-G_3_-NGR solution (0.1 mL) with 0.9 mL of human serum at 37 °C or 0.1 mL of the saline at room temperature with gentle shaking. The radiochemical purity was measured at various time points (1, 2, and 4 h).

### 3.6. Cell Culture and Animal Model

The HT-1080 human fibrosarcoma and HT-29 human colon adenocarcinoma cells were maintained in high glucose DMEM culture medium supplemented with 10% (v/v) fetal bovine serum (Life Technologies, Grand Island, NY, USA), 1% L-glutamine, and 1% mycillin (Beyotime, Haimen, Jiangsu Province, China) in a humidified atmosphere of 5% CO_2_ at 37 °C. HT-1080 and HT-29 tumor xenografts were established by subcutaneously injecting 0.1 mL of tumor cell suspension (5 × 10^6^ cells) into the right upper flank of female nude BALB/c mice (4–6 weeks). When tumors reached 500–1000 mm^3^ in volume, mice were used for microPET imaging and biodistribution studies. All animal studies were approved by the Clinical Center at the Fourth Military Medical University.

### 3.7. Cell Binding Assay

*In vitro* CD13 receptor binding affinity and specificity of NOTA-G_3_-NGR in HT-1080 cells were assessed using a cell-based competitive assay. ^125^I-labeled linear NGR peptide (sequence H-Tyr-Gly-Gly-Cys-Asn-Gly-Arg-Cys-OH) was prepared using the Iodogen method and used as the radioligand for CD13. The non-radiolabeled NOTA-G_3_-NGR peptide (concentrations at a range from 10^−12^ to 10^−5^ M) was co-incubated with ^125^I-NGR in HT-1080 cells. After washing with ice-cold binding buffer three times, the HT-1080 cells were lysed in 200 µL of lysis buffer. The cell-associated radioactivity was then measured using a gamma counter (Beijing PET CO., Ltd., Beijing, China). The IC_50_ (50% inhibitory concentration) value was obtained by fitting the data using nonlinear regression with GraphPad Prism (GraphPad Software, San Diego, CA, USA). Experiments were performed in triplicate.

### 3.8. Cell Uptake and Efflux

CD13-positive HT-1080 or CD13-negative HT-29 cells were seeded into a 48-well plate at a density of 2.5 × 10^5^ cells per well and grown overnight. The cells were then incubated with ^68^Ga-NOTA-G_3_-NGR (~18 kBq/well) at 37 °C for 15, 30, 60, and 120 min. After incubation, tumor cells were washed three times with chilled PBS and harvested by trypsinization with 0.25% trypsin/0.02% EDTA (Beyotime). Cell suspensions were collected and measured in a gamma counter (Beijing PET CO., Ltd.). Cell uptake data was presented as percentage of total input radioactivity after decay correction. Experiments were performed twice with triplicate wells.

For efflux studies, HT-1080 or HT-29 tumor cells were incubated with ^68^Ga-NOTA-G_3_-NGR (~18 kBq/well) for 2 h at 37 °C to allow internalization. Cells were then washed twice with PBS, and incubated with cell culture medium for 15, 30, 60, and 120 min. After washing three times with PBS, cells were harvested by trypsinization with 0.25% trypsin/0.02% EDTA (Beyotime). Cell suspensions were collected and measured in a gamma-counter (Beijing PET CO., Ltd.). Efflux data was presented as percentage of total input radioactivity after decay correction. Experiments were conducted twice with triplicate wells.

### 3.9. MicroPET Imaging

MicroPET scans were performed using Mediso NanoPET/CT scanner (Mediso, Budapest, Hungary). HT-1080 (CD13-positive) or HT-29 (CD13-negative) tumor bearing nude mice (*n* = 5/group) under isoflurane anesthesia were intravenously injected with ~3.7 MBq of ^68^Ga-NOTA-G_3_-NGR. The 30-min continuous dynamic PET scan started at 1 min pi. Ten-minute static scans were acquired at 0.5, 1, and 2 h pi. Prior to each PET imaging, helical CT scan was acquired. PET and CT fused images were obtained using the automatic image fusion software (Mediso Medical Imaging Systems, Budapest, Hungary). For blocking experiment, mice bearing HT-1080 tumors (*n* = 5/group) were co-injected with 20 mg/kg NOTA-G_3_-NGR and ~3.7 MBq of ^68^Ga-NOTA-G_3_-NGR. The accumulation of radioactivity in tumor and other tissues was obtained from the mean values measured by the ROIs, and then converted to % ID/g.

### 3.10. Biodistribution Study

The biodistribution study was carried out using nude mice bearing HT-1080 or HT-29 tumor. Each mouse was injected with 370 kBq of ^68^Ga-NOTA-G_3_-NGR via tail vein. The mice were euthanized by cervical dislocation at 60 min after injection of ^68^Ga-NOTA-G_3_-NGR. Tumor, blood, muscle, and major organs (heart, stomach, lung, spleen, liver, pancreas, kidneys, and intestine) were harvested, weighed wet, and measured for radioactivity in a gamma counter (Beijing PET CO., Ltd.). The radioactivity was decay corrected to the time of injection. The biodistribution results were presented as %ID/g. The uptake values were obtained as mean %ID/g ± SD (*n* = 5/group).

### 3.11. Statistical Analysis

Quantitative data were expressed as mean ± SD. Means were compared using one-way ANOVA and Student’s *t* test. *p* values < 0.05 were considered statistically significant.

## 4. Conclusions

Tumors growth and progression are angiogenesis-depended in general [26]. Noninvasive imaging of tumor angiogenesis would eventually lead to effective anti-angiogenesis treatment. Unlike conventional imaging modalities, such as computed tomography (CT), which mainly provide detailed anatomical images, PET can measure biochemical and physiological aberrations that occur prior to macroscopic anatomical signs of a disease, such as cancer [4]. PET requires administration of molecular probes in a tested subject in order to acquire the imaging signals generated from molecular probes which are labeled with positron-emitting radionuclides [2]. However, the most commonly used PET probe (^18^F-FDG) is not a target-specific probe, ^18^F-FDG is not able to directly and accurately assess tumor angiogenesis. Therefore, the development of PET probes which can specifically target to tumor angiogenesis is highly demanded.

CD13 receptor which overexpresses on tumor neovasculature is one of attractive biological targets. The aim of this study is to develop a ^68^Ga-labeled NGR-containing peptide for noninvasively imaging CD13 expression *in vivo* by PET. In our previous study, we have successfully developed a CD13-specific ^64^Cu-DOTA-NGR2 probe. Moving the radionuclide from ^64^Cu to ^68^Ga for PET study, we selected ^68^Ga-NOTA system whose stability has been shown to be higher than that of ^68^Ga-DOTA or ^64^Cu-DOTA [25]. In fact, much lower liver uptake of ^68^Ga-NOTA-G_3_-NGR (<3%ID/g) was observed in this study as compare to that of ^64^Cu-DOTA-NGR2 (>8%ID/g) [8] at the same imaging time point (1 h pi). This observation may presumably be due to the less demetalation and subsequent radioactivity accumulation in liver for ^68^Ga-NOTA system *versus* ^64^Cu-DOTA system. In addition, significantly higher tumor uptake of ^68^Ga-NOTA-G_3_-NGR in CD13-positive HT-1080 tumors *versus* CD13-negative HT-29 tumors, and effective blocking in HT-1080 tumors were observed, demonstrating that ^68^Ga-NOTA-G_3_-NGR is indeed a CD13-specific PET probe. Nonetheless, an appropriate compartment model may be set up to more accurately analyze specific and non-specific binding of radiolabeled probe after collecting blood samples at various imaging time points. Moreover, favorable pharmacokinetics of ^68^Ga-NOTA-G_3_-NGR warrants further translational studies for imaging CD13-positive tumors by PET.

## Supplementary Materials

Supplementary materials can be accessed at: http://www.mdpi.com/1420-3049/19/8/11600/s1.

## Supplementary Files

Supplementary File 1
