# Supplementary material for: 68Ga-Labeled Cyclic NGR Peptide for MicroPET Imaging of CD13 Receptor Expression
Source: Molecules. 2014 Aug 5;19(8):11600–12. doi: 10.3390/molecules190811600 (PMC6271277; doi:10.3390/molecules190811600)

Supplementary Information

1. HPLC Method and Analysis of NOTA-G_3-NGR Peptide

Analytical reversed phase HPLC for NOTA-G_3-NGR peptide was performed on a Thermo UltiMate 3000 HPLC system. A Dionex Acclaim120 C18 reversed phase analytical column (5 µm, 250 × 4.6 mm) was used. The flow rate was 1 mL/min with the mobile phase starting from 100% solvent A (0.1% TFA in water) to 70% solvent A and 30% solvent B (0.1% TFA in acetonitrile) at 20 min. The UV absorbance was monitored at 214 nm. The retention time (R*t*) of NOTA-G_3-NGR was determined to be 17.4 min.

Figure S1. HPLC profile of NOTA-G_3-NGR peptide (UV @ 214 nm).

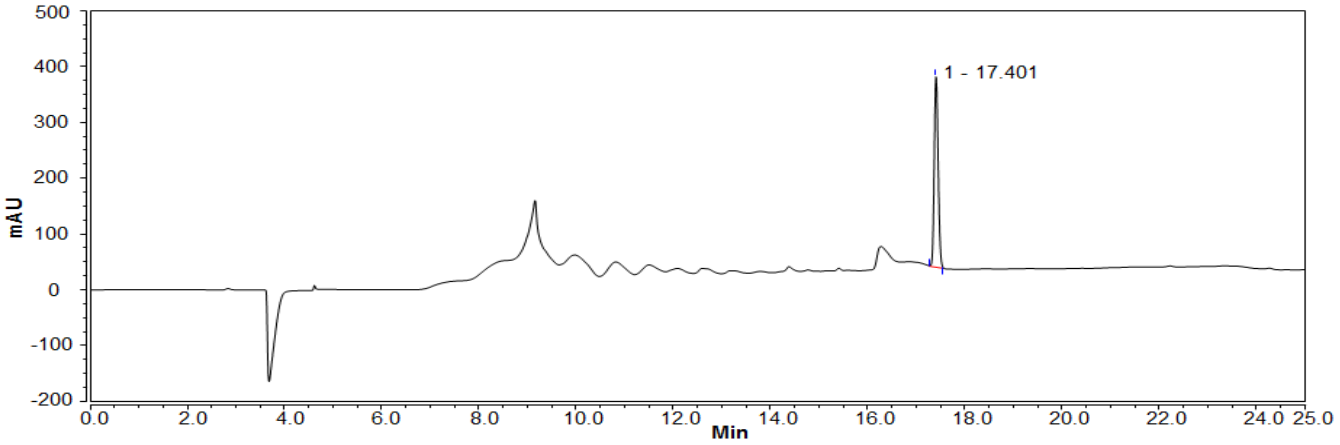

2. Mass Spectrum

Figure S2. Mass spectrum of NOTA-G_3-NGR peptide.

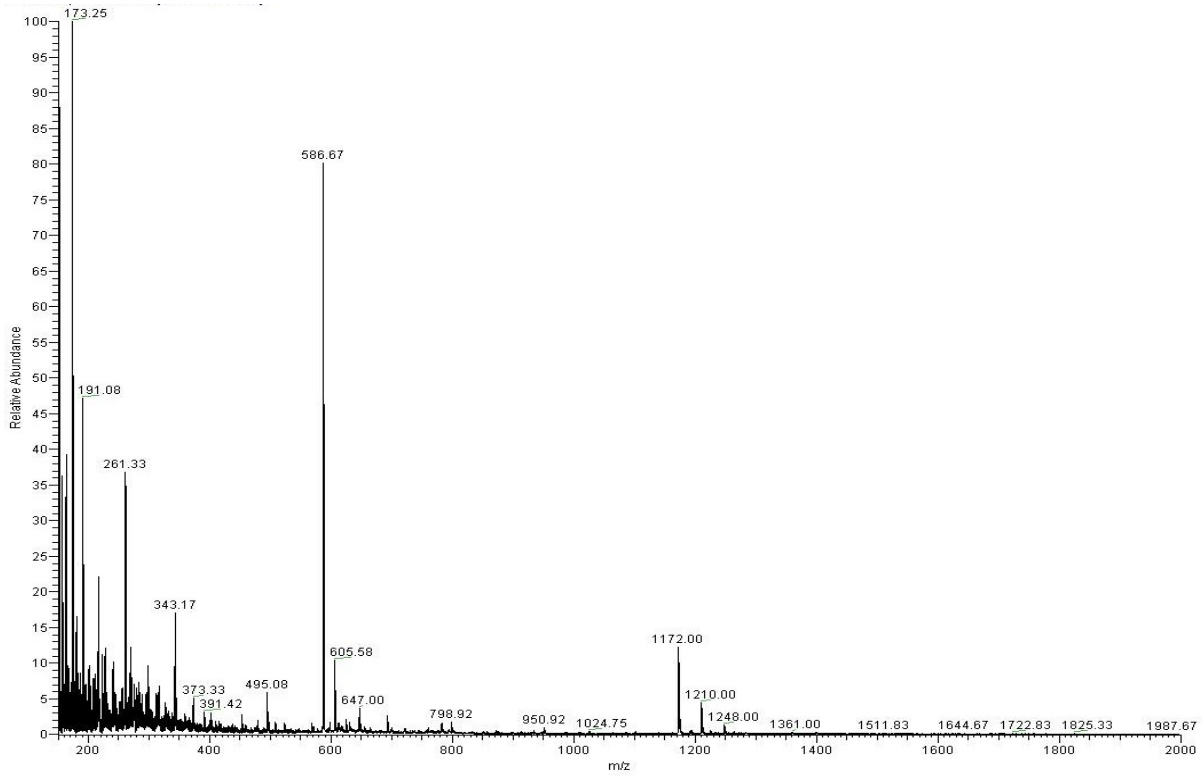

3. Determination of Radiochemical Purity (RCP)

The RCP of ^{68}Ga-NOTA-G_3-NGR was determined using a Miniscan TLC scanner (Bioscan, Washington, DC, USA) with silica gel-coated plastic sheets (Polygram SIL G, Macherey-Nagel). Sodium citrate (0.1 M, pH = 5) was used as the developing solvent. The radiolabeled peptide remained in the origin, whereas free ^{68}Ga moved to the solvent front (R_f = 0.8–1.0).

Figure S3. Thin layer chromatography spectrum. (**a**) Radio-TLC of ^{68}Ga-NOTA-G_3-NGR; (**b**) Free ^{68}Ga.

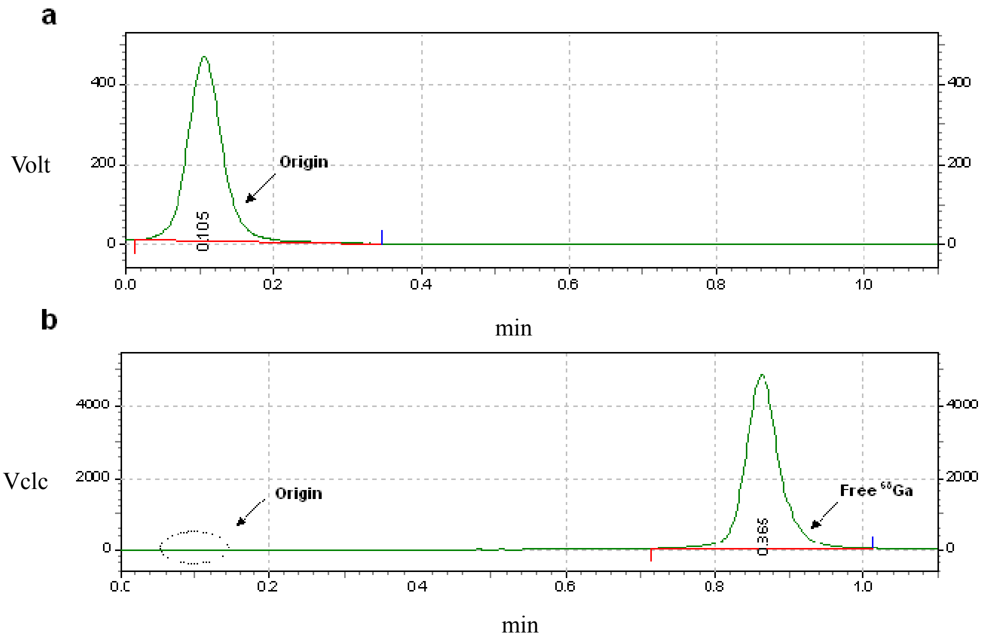

4. Western Blot Analysis

Cell extracts were obtained by lysing cells in cell lysis buffer (Beyotime, Haimen, Jiangsu Province, China) supplemented with protease inhibitors (Roche, Mississauga, Canada). The extractions were then centrifuged at 12,000 rpm for 10 min at 4 °C. The protein concentration of supernatant was determined using the Bradford Protein Assay Kit (Beyotime, China). Cell extractions (40 µg of protein) were loaded on SDS-PAGE gels and transferred to a PVDF membrane (Life Technologies, Grand Island, NY, USA). Membranes were blocked with 5% non-fat dry milk and incubated with anti-CD13 monoclonal antibody (1:100; Abcam, Cambridge, MA, USA) overnight and peroxidase-conjugated secondary antibody (1:400; Life Technologies, Grand Island, NY, USA) for 2 h according to the manufacturer's instructions.

The antigen–antibody complexes on the membranes were visualized with ECL Western Blotting Detection System (Thermo Fisher Scientific, IL, USA) with ChemiDOC XRS+ (Bio-Rad, Hercules, CA, USA). Beta-actin was detected with anti-*β*-actin as an internal loading control.

Figure S4. (**a**) Representative western blot analyses of CD13 receptor expression (150 kDa) in HT-1080 and HT-29 cells. *β*-Actin (44 kDa) was used as a loading control; (**b**) CD13/β-actin ratios in HT-1080 and HT-29 cells. The data demonstrated that CD13 receptors are overexpressed in HT-1080 cells, but not in HT-29 cells.

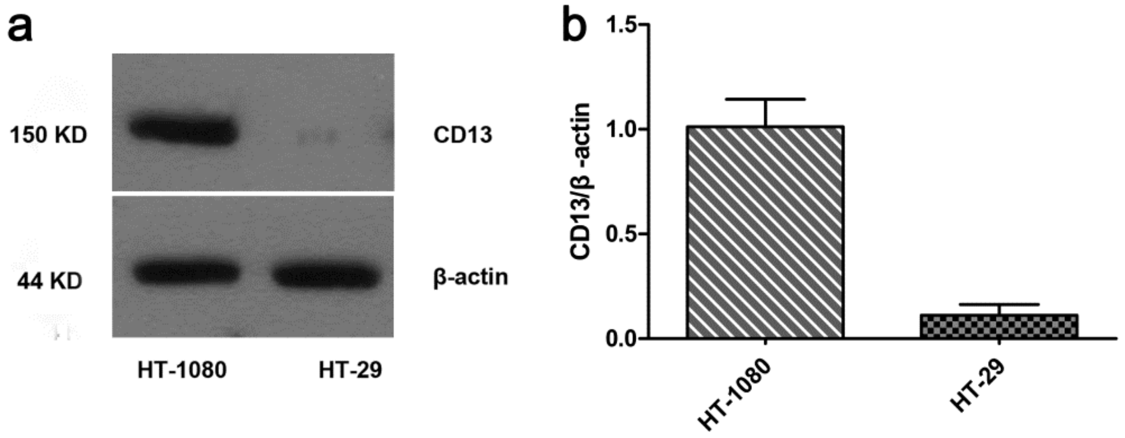

5. H&E and Immunohistochemistry Staining of CD13 Receptors in HT-1080 Tumors

After microPET imaging, mice were euthanized, and HT-1080 tumors were dissected and fixed with 10% formalin. Routine hematoxylin and eosin staining (H&E) and immunohistochemistry staining of CD13 receptors in HT-1080 tumors were carried out. The dilution of anti-CD13 monoclonal antibody was 1:100. Tumor slices were observed under an Olympus microscope and the images were captured. Brown-colored staining in the cell membrane was considered as a positive result of CD13 expression.

Figure S5. (**a**) H&E staining; (**b**) Immunohistochemistry staining of CD13 receptors in HT-1080 tumors. The brown color indicates CD13 expression. The images were taken at 200× magnification.

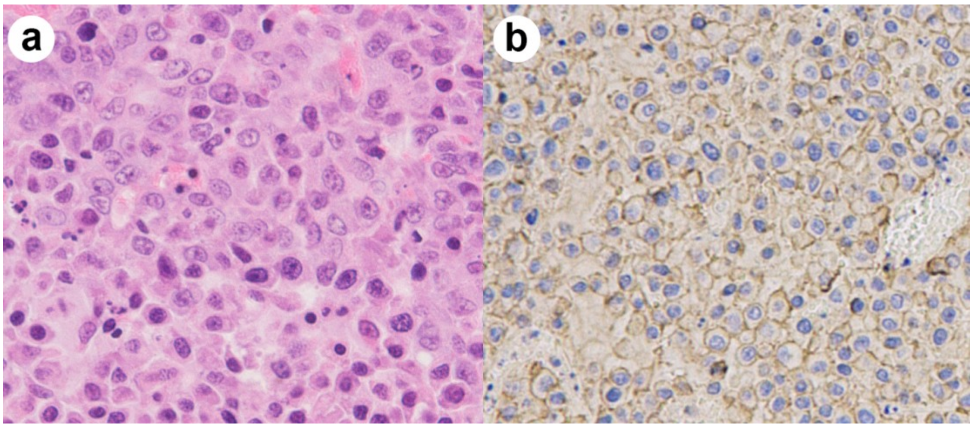

Supplement: Supplementary File 1 [file molecules-19-11600-s001.pdf]
